# Cultivation Area Affects the Presence of Fungal Communities and Secondary Metabolites in Italian Durum Wheat Grains

**DOI:** 10.3390/toxins12020097

**Published:** 2020-02-03

**Authors:** Giovanni Beccari, Antonio Prodi, Maria Teresa Senatore, Virgilio Balmas, Francesco Tini, Andrea Onofri, Luca Pedini, Michael Sulyok, Luca Brocca, Lorenzo Covarelli

**Affiliations:** 1 Department of Agricultural, Food and Environmental Sciences, University of Perugia, Borgo XX Giugno, 74, 06121 Perugia, Italy; giovanni.beccari@unipg.it (G.B.); francesco.tini@unipg.it (F.T.); andrea.onofri@unipg.it (A.O.); lucapedini92@gmail.com (L.P.); or; 2 Department of Agricultural and Food Sciences, Alma Mater Studiorum University of Bologna, Viale G. Fanin, 44, 40127 Bologna, Italy; mariateresa.senatore@unibo.it; 3 Department of Agriculture, University of Sassari, Via E. De Nicola, 9, 07100 Sassari, Italy; balmas@uniss.it; 4 Department of Agrobiotechnology (IFA-Tulln), University of Natural Resources and Applied Life Sciences, Vienna (BOKU), Konrad Lorenz Strasse, 20, A-3430 Tulln, Austria; michael.sulyok@boku.ac.at; 5 Research Institute for Geo-Hydrological Protection, National Research Council, Via della Madonna Alta, 126, 06128 Perugia, Italy; luca.brocca@irpi.cnr.it

**Keywords:** *Fusarium* head blight, *Triticum turgidum* subsp. *durum*, mycotoxins, cereals, wheat

## Abstract

In this study, durum wheat kernels harvested in three climatically different Italian cultivation areas (Emilia Romagna, Umbria and Sardinia) in 2015, were analyzed with a combination of different isolation methods to determine their fungal communities, with a focus on *Fusarium* head blight (FHB) complex composition, and to detect fungal secondary metabolites in the grains. The genus *Alternaria* was the main component of durum wheat mycobiota in all investigated regions, with the Central Italian cultivation area showing the highest incidence of this fungal genus and of its secondary metabolites. *Fusarium* was the second most prevalent genus of the fungal community in all cultivation environments, even if regional differences in species composition were detected. In particular, Northern areas showed the highest *Fusarium* incidence, followed by Central and then Southern cultivation areas. Focusing on the FHB complex, a predominance of *Fusarium poae*, in particular in Northern and Central cultivation areas, was found. *Fusarium graminearum*, in the analyzed year, was mainly detected in Emilia Romagna. Because of the highest *Fusarium* incidence, durum wheat harvested in the Northern cultivation area showed the highest presence of *Fusarium* secondary metabolites. These results show that durum wheat cultivated in Northern Italy may be subject to a higher FHB infection risk and to *Fusarium* mycotoxins accumulation.

## 1. Introduction

With a production of about 4.4 million tons and a cultivated area of 1.3 million hectares in 2015, Italy is one of the most important countries in the world for durum wheat (*Triticum turgidum* subsp. *durum* (Desf.) Husn.) cultivation [1,2]. In fact, durum wheat is the main crop in many regions of the peninsula [2] and in the last decades, its cultivation expanded from the “typical” Southern Italian areas to Central and Northern parts of the country. For example, in Umbria (Central Italy) and in Emilia Romagna (Northern Italy), durum wheat production doubled during the 2005–2015 period [2]. In detail, in 2015 about 65% of durum wheat national production was obtained in Southern regions, almost 23% in Central regions, and the remaining 12% in Northern regions [2]. Italian durum wheat is mainly used for pasta production. In 2014, Italy was the country with the highest pasta production and consumption worldwide, with about 3.4 million tons and 25 kg per capita, respectively [3]. For these reasons, durum wheat plays a key role in the Italian and European agri-food compartment. Therefore, the maintenance of a high qualitative standard is an essential aspect of its cultivation across the country. However, several fungal microorganisms, being able to infect/contaminate durum wheat kernels in the field, represent a serious threat to grain production and quality. Additionally, the aforementioned expansion of durum wheat cultivation from Southern to Northern Italian areas has significantly increased this risk, due to more favorable climatic conditions to diseases [4,5]. The fungal community colonizing durum wheat grains mainly consists of mycotoxigenic genera, principally *Alternaria* and *Fusarium* [6,7,8,9]. Most of the species belonging to these genera are able to biosynthesize mycotoxins, secondary fungal metabolites having strong acute and chronic adverse effects on animals and humans [10]. Thus, the presence of toxigenic mycobiota in durum wheat kernels represents a non-negligible risk factor for consumers’ health [11] because of the classification of some fungal secondary metabolites as carcinogens or possible carcinogens by the International Agency for Research on Cancer (IARC). Therefore, the European Union (EU) has established maximum or recommended levels for several mycotoxins in various food matrixes, such as raw durum wheat and some derived products such as pasta [12,13]. For other compounds, the European Food Safety Authority (EFSA) performed risk assessments and gave scientific opinions [14,15].

Among the various fungal microorganisms, those belonging to the genus *Fusarium* are particularly dangerous. They are the causal agents of *Fusarium* head blight (FHB) a disease capable of strongly impairing not only crop yield but also its quality. In fact, FHB is caused by many distinct species belonging to multiple *Fusarium* species complexes [16] that, in addition to being able to infect and damage wheat heads in the field, may biosynthesize a wide range of toxic secondary metabolites. Since durum wheat is more susceptible to FHB than soft wheat (*Triticum aestivum* L.) [17,18], *Fusarium* infections and mycotoxin contaminations are of particular concern in Italy [19]. Moreover, FHB pathogens negatively affect durum wheat quality as they may damage the protein fraction of the kernels [20,21]. Among FHB causal agents, *Fusarium graminearum* is globally considered as the most important, because of its widespread incidence and aggressiveness [22] as well as because it is the main producer of type B trichothecenes such as deoxynivalenol (DON) and nivalenol (NIV) [23]. A similar toxigenic profile also characterizes *Fusarium culmorum*, an important FHB agent in Southern Mediterranean areas [24]. In recent years, other *Fusarium* species such as *Fusarium poae* and *Fusarium avenaceum* increased their importance in the FHB community in many wheat-cultivation areas worldwide [25,26,27,28,29,30]. In particular, in certain Italian regions and cultivation seasons, these two species were found to be the most prominent members of the FHB complex of wheat, even more present than *F. graminearum* [7,8,18,31,32,33]. *F. avenaceum* is mainly able to biosynthesize depsipetides, such as enniatin analogues (ENs) and moniliformin (MON) [34,35]. *F. poae,* in addition to NIV, produces depsipeptides such as beauvericin (BEA) [34,35]. Type A trichothecenes, such as T-2 and HT-2 toxins [35], are mainly produced by *Fusarium sporotrichioides* and *Fusarium langsethiae* [36,37]. The isolation of these last two species from the grains has been demonstrated to be difficult [36,38,39] and their presence might consequently be often underestimated. Other *Fusarium* species may also be detected with a low incidence in wheat grains. FHB complexity is exacerbated by the fact that, in addition to well-known mycotoxins, a wide range of other secondary metabolites produced by *Fusarium* species and having an unknown impact on health, might also potentially occur in the kernels [7,40,41].

The FHB complex composition as well as the type and quantity of mycotoxins in the grain, strongly depend on climatic factors such as temperature, humidity and rainfall, especially those occurring during crop anthesis [42,43,44,45]. Thus, seasonal patterns usually show a strong influence on the incidence of the different *Fusarium* species and of their mycotoxins in the grains [46,47]. In addition, agronomic factors such as crop rotations, crop management systems [27,47,48,49], deployed varieties [50] and the use of fungicides [51] may affect FHB complex composition and the presence of secondary metabolites. One or more of these factors might potentially be the reason for regional differences within the FHB complex as well as for the presence of the mycotoxins associated with wheat grains in different world cultivation areas [30,52,53,54,55,56]. For example, in Italy, FHB incidence and DON occurrence usually increase from Southern to Northern regions [18,19].

Given this diversity, a better understanding of the regional composition of the FHB complex as well as of mycotoxin occurrence are critical to understanding the effective suitability of a certain geographic area for producing high-quality durum wheat and for setting up effective disease and mycotoxin integrated management strategies in different cultivation areas [52].

Therefore, in the present study, durum wheat kernels harvested in three climatically different Italian regions (Emilia Romagna, Umbria and Sardinia), representative of three different cultivation areas (North, Center and South, respectively) were analyzed to: (1) determine the mycobiota infecting the grains with two different isolation methods (potato dextrose agar, PDA and deep-freezing blotter, DFB); (2) molecularly determine FHB complex composition; (3) quantify the main *Fusarium* species in the grains by quantitative real-time polymerase chain reaction (qPCR); (4) detect a wide range of fungal secondary metabolites in the grains by liquid chromatography tandem mass spectrometry (LC-MS/MS). The main objective of this investigation was to determine the importance of different cultivation areas in affecting the presence of fungal communities, including FHB causal agents, as well as of secondary metabolites in Italian durum wheat grains.

## 2. Results

### 2.1. Mycobiota Composition

**Figure 1:**
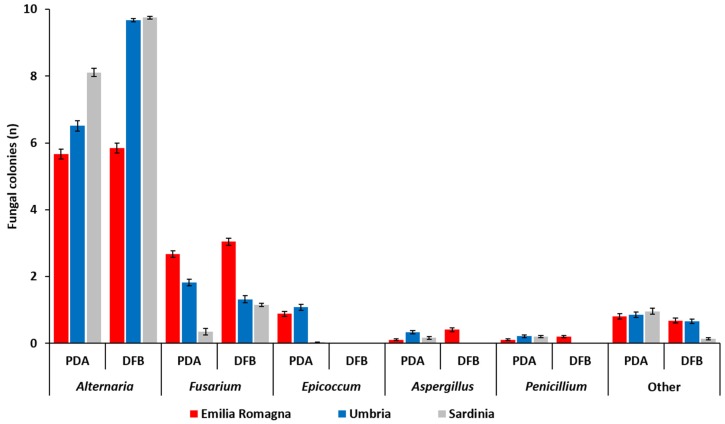
Average number of fungal colonies (*n*) per durum wheat sample belonging to different fungal genera as visually and microscopically assessed after their development from durum wheat kernels collected in three different Italian regions (Emilia Romagna, Umbria, Sardinia) with two different isolation methods (potato dextrose agar, PDA; deep-freezing blotter, DFB). Columns represent the average (±standard error, SE) number of colonies belonging to different fungal genera developed from 10 analyzed durum wheat samples per each region and with each method.

The number (*n*) of fungal colonies belonging to the genera *Alternaria*, *Fusarium*, *Epicoccum*, *Aspergillus* and *Penicillium* developing after 5 (PDA) or 7 (DFB) days of incubation from grains sampled in each of the three Italian regions is shown in Figure 1 and detailed in Table S1. Fungi that were not included in the aforementioned genera were classified as “other” (Figure 1, Table S1).

The fungal colonies belonging to the genus *Alternaria* showed a significantly higher presence (*p* ≤ 1 × 10^−4^) within the fungal community developed from durum wheat grains following both PDA and DFB isolations in all the surveyed regions. In addition, with the exception of samples from Emilia Romagna (*p* = 0.41), the number of *Alternaria* colonies recovered with the DFB was significantly higher (*p* ≤ 1 × 10^−4^) than those detected on PDA (Figure 1, Table S1). *Alternaria* presence in the Emilia Romagna samples was significantly lower (*p* ≤ 1 × 10^−4^) than that recovered in the Sardinian and in the Umbrian ones with the DFB and on PDA. Conversely, in the Sardinian samples, the isolates belonging to this genus were more frequent than in the Umbrian ones, even if only on PDA (*p* = 1 × 10^−4^) and not with the DFB (*p* = 0.34) (Figure 1, Table S1).

The genus *Fusarium* was the second component of the durum wheat grain mycobiota following both DFB (*p* ≤ 1 × 10^−4^) and PDA (*p* ≤ 0.04) isolations in all surveyed areas. Significant differences between the two isolation methods were recorded in Sardinia, where *Fusarium* colonies were significantly higher with the DFB than on PDA (*p* = 1 × 10^−4^) as well as in Umbria, where *Fusarium* colonies grown on PDA were significantly higher than those isolated with the DFB (*p* = 0.002) (Figure 1, Table S1). In Emilia Romagna, the presence of *Fusarium* colonies developing from the grains on PDA was significantly higher than that of the Umbrian samples (*p* = 1 × 10^−4^), in which they were significantly higher than in the Sardinian ones (*p* = 1 × 10^−4^). Similar results were also observed following DFB isolations (*p* ≤ 1 × 10^−4^), even if in this case Umbria and Sardinia did not significantly differ from each other (*p* = 0.24) in terms of isolated *Fusarium* colonies.

In addition to the two most frequently recovered fungal genera (*Alternaria* and *Fusarium*), the mycobiota of durum wheat grains harvested in the three investigated Italian regions was also composed of other fungal genera, such as *Epicoccum*, *Aspergillus* and *Penicillium*. The colonies belonging to the genus *Epicoccum* developed only on PDA while they were not isolated with the other method (Figure 1). In addition, in Emilia Romagna and Umbria the incidence of *Epicoccum* spp. on PDA was significantly higher than that recorded in Sardinia (*p* ≤ 1 × 10^−4^). With the exception of Emilia Romagna samples, also colonies of *Aspergillus* and *Penicillium* were obtained only on PDA and not with the DFB. On PDA, the presence of *Aspergillus* was higher in the Umbrian samples (*p* ≤ 0.01). On the same medium, no significant differences between different regions (*p* ≥ 0.07) in terms of number of developed *Penicillium* colonies were found.

### 2.2. Fusarium Head Blight (FHB) Complex Composition

**Figure 2:**
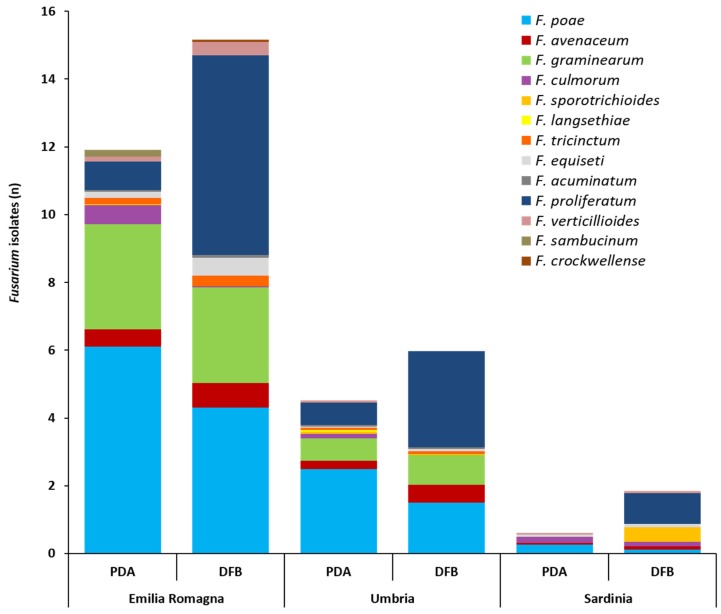
Average number of isolates (*n*) per durum wheat sample belonging to the different *Fusarium* species as identified by partial *translation elongation factor 1α* sequencing after their isolation from durum wheat kernels collected in three different Italian regions (Emilia Romagna, Umbria, Sardinia) with two isolation methods (potato dextrose agar, PDA; deep-freezing blotter, DFB). Columns represent the *Fusarium* community composition expressed as the average number of isolates of different species developed from 10 durum wheat samples per each region and with each method.

With regard to the total *Fusarium* isolates developed from durum wheat with the two isolation methods and successively identified by partial *translation elongation factor 1α* (*tef1α*) region sequencing, significant differences between cultivation regions were detected. In detail, the average number of total *Fusarium* isolates infecting the grains followed the statistically significant gradient: Emilia Romagna >> Umbria > Sardinia. This was observed both for PDA (11.8, 4.7, 0.74, respectively) (*p* ≤ 0.002) and for the DFB method (15.2, 6.10, 1.96, respectively) (*p* ≤ 0.01). Even if in the three surveyed regions the number of *Fusarium* isolates recovered with the DFB was higher than that obtained on PDA, no significant differences between the two methods in any of the investigated areas (*p* ≥ 0.1) were found. The number (*n*) of *Fusarium* isolates ascribable to the different species is shown in Figure 2 and detailed in Table S2.

The *Fusarium* community isolated on PDA and with the DFB method from the kernels collected across Emilia Romagna was composed of 11 different species (Figure 2, Table S2). On PDA, *F. poae* was the most frequent species (6.1) (*p* ≤ 0.001) followed by *F. graminearum* (3.0) (*p* ≤ 7 × 10^−4^). Eight more species (*Fusarium proliferatum*, *F. culmorum*, *F. avenaceum*, *Fusarium equiseti*, *Fusarium sambucinum*, *Fusarium tricinctum*, *Fusarium verticillioides* and *Fusarium acuminatum*) (Figure 2, Table S2) were isolated from the kernels on PDA, showing a significantly lower presence than *F. poae* and *F. graminearum* (*p* ≤ 7 × 10^−4^) (Table S2). With DFB, *F. proliferatum* (5.98) and *F. poae* (4.33) showed the significantly highest presence (*p* ≤ 1 × 10^−4^). These species were followed also in this case by *F. graminearum* (2.82), which was significantly lower than *F. proliferatum* (*p* = 7 × 10^−4^) but not than *F. poae* (*p* = 0.07). The other six species (*F. avenaceum*, *F. equiseti*, *F. verticillioides*, *F. tricinctum*, *F. acuminatum* and *Fusarium crockwellense*) were significantly lower than the previous three species (*p* ≤ 2 × 10^−4^). Differences in recovering *Fusarium* species between the two isolation methods in terms of number of *Fusarium* isolates were recorded only for *F. culmorum* and *F. proliferatum*. In detail, *F. culmorum* showed a significantly higher presence on PDA (0.56) than with DFB (nd) (*p* = 0.03), while, *F. proliferatum* showed a significantly higher presence with DFB (5.98) than on PDA (0.84) (*p* = 1 × 10^−4^).

The *Fusarium* community isolated on PDA and with DFB from durum wheat kernels collected in Umbria was composed of 11 different species (Figure 2, Table S2). On PDA, *F. poae* showed a significantly higher presence (2.53) (*p* ≤ 0.001) followed by *F. proliferatum* (0.67) and *F. graminearum* (0.66), which showed a non-significant difference (*p* = 0.97) with each other. The other seven *Fusarium* species (*F. avenaceum*, *F. culmorum*, *F. langsethiae*, *F. sporotrichioides*, *F. tricinctum*, *F. acuminatum* and *F. verticillioides*) isolated on PDA showed a similar presence (*p* ≥ 0.29) and only *F. aveanceum* and *F. culmorum* were not significantly different from *F. proliferatum* and *F. graminearum* (*p* ≥ 0.06). With DFB, *F. proliferatum* (2.82) was the most isolated species even if it was not significantly different from *F. poae* (1.55) (*p* = 0.06). These two species were followed by *F. graminearum* (0.87), which was significantly lower than *F. proliferatum* (*p* = 0.001) only. The other four *Fusarium* species (*F. avenaceum*, *F. tricinctum*, *F. equiseti* and *F. acuminatum*) showed a similar presence. *F. avenaceum* (0.53) was the only species not being significantly different from *F. graminearum* (*p* = 0.37). In addition, in this case, only *F. proliferatum* showed a significantly higher presence with the DFB (2.82) than on PDA (0.67) (*p* = 4 × 10^−4^).

The *Fusarium* community isolated on PDA and with the DFB method from durum wheat kernels collected in Sardinia was composed of seven different species (Figure 2, Table S2). On PDA, no significant differences were recorded among the five different species isolated (*F. poae*, *F. culmorum*, *F. graminearum*, *F. proliferatum*, *F. avenaceum*) (*p* ≥ 0.24). With DFB, *F. proliferatum* showed a significantly higher presence (0.92) (*p* ≤ 0.02) than the other five species (*F. culmorum*, *F. poae*, *F. avenaceum*, *F. equiseti*, *F. verticillioides*), with the exception of *F. sporotrichioides* (0.47) (*p* = 0.27). Differences between the two isolation methods in terms of number of *Fusarium* isolates were recorded not only for *F. proliferatum* but also for *F. sporotrichioides*. Both species showed a significantly higher presence with DFB than on PDA (*p* = 0.005 and 0.04, respectively).

Significant differences (*p* ≤ 0.04) between cultivation regions in terms of single species presence (Table S2) were also detected. In detail, *F. poae* followed the significant gradient Emilia Romagna > Umbria > Sardinia (both for PDA and DFB); *F. graminearum* followed the significant gradient Emilia Romagna > Umbria >> Sardinia (absent) (both for PDA and DFB); *F. avenaceum* followed the significant gradient Emilia Romagna ≥ Umbria ≥ Sardinia (only for PDA); *F. proliferatum* followed the significant gradient Emilia Romagna ≥ Umbria > Sardinia (both for PDA and DFB).

### 2.3. Fungal Biomass Accumulation

R^2^ values calculated from the linear equations of the six standard curves were 0.99 for all *Fusarium* species, with the exception of *F. avenaceum* whose R^2^ value was 0.97. Reaction efficiencies obtained from the linear equations of the six standard curves were 1.97 for *F. graminearum* and *F. avenaceum*, 1.94 for *F. poae*, 1.98 for *F. langsethiae*, 1.99 for *F. culmorum* and 1.96 for *F. sporotrichioides*. Dissociation curve analysis showed specific amplification products in the presence of pure fungal DNA (standard curves) and in the presence of DNA of the six investigated *Fusarium* species (samples). No target amplification was detected in negative controls. For these reasons, cycle threshold (Ct) values used to quantify fungal biomass were those for which dissociation curve analysis showed the presence of specific amplification products.

**Figure 3:**
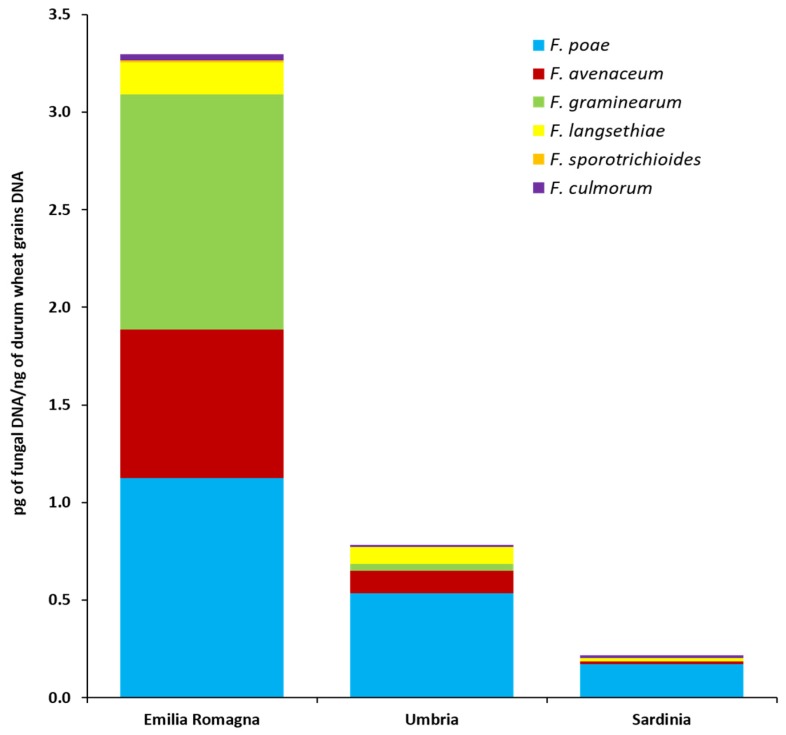
Fungal biomass of six *Fusarium* species as detected by quantitative real-time polymerase chain reaction (PCR) assays in durum wheat kernels collected in three different Italian regions (Emilia Romagna, Umbria, Sardinia). Columns represent *Fusarium* community composition, expressed as the average of pg of each analyzed fungal species DNA/ng of durum wheat grains DNA in 10 analyzed samples per each region.

The fungal biomass of six *Fusarium* species (pg of fungal DNA/ng durum wheat grains DNA) as quantified by qPCR directly in durum wheat grains collected in three different Italian regions (Emilia Romagna, Umbria, Sardinia) is shown in Figure 3 and detailed in Table S3.

In the durum wheat grains harvested in Emilia Romagna, all six analyzed species were detected. *F. poae* was present in all Emilia Romagna samples and *F. graminearum* and *F. avenaceum* were both detected in 9 out of 10 Emilia Romagna samples. In addition, *F. langsethiae*, *F. culmorum* and *F. sporotrichioides* were present in 4, 3 and 1 Emilia Romagna samples, respectively. *F. poae* showed the highest fungal biomass levels, followed by *F. graminearum* and *F. avenaceum* even if no significant differences between these three species were recorded (*p* ≥ 0.20). Similarly, *F. langsethiae*, *F. culmorum* and *F. sporotrichioides* were not significantly different from each other (*p* ≥ 0.15), but they showed significantly lower fungal biomass levels than the previous ones (*p* ≤ 0.03).

In the durum wheat grains harvested in the Umbria region, all the species analyzed by qPCR, with the exception of *F. sporotrichioides*, were detected. *F. poae*, present in all the analyzed samples (*n* = 10), showed a significantly higher biomass level (*p* ≤ 0.01) than those of the other four species. No significant differences (*p* ≥ 0.16) between *F. avenaceum*, *F. graminearum*, *F. langsethiae* and *F. culmorum* fungal biomasses were detected. These species were present in 4 (*F. avenaceum* and *F. langsethiae*), 2 (*F. graminearum*) and 1 (*F. culmorum*) out of 10 durum wheat grains samples, respectively.

In the durum wheat grains collected across Sardinia, 4 out of 6 analyzed species were detected. *F. poae* was present in 6 out of 10 samples. *F. culmorum* and *F. langsethiae* were both present in 2 samples, while, *F. avenaceum* was present in only one sample. No significant differences (*p* ≥ 0.10) in fungal biomass accumulation for these four species were detected in the Sardinian samples. No *F. graminearum* and *F. sporotrichioides* were detected in the durum wheat grains collected in this area.

Significant differences between cultivation areas in terms of fungal accumulation in the grains (Figure 3 and Table S3) were also detected for *F. poae* and *F. avenaceum*. In detail, *F. poae* followed the significant gradient (*p* ≤ 0.04) Emilia Romagna >> Umbria > Sardinia; *F. avenaceum* followed the significant gradient (*p* ≤ 0.001) Emilia Romagna > Umbria = Sardinia. For *F. graminearum* and *F. langsethiae*, although their presence was higher in the Emilia Romagna region, no significant differences were recorded between the different surveyed areas (*p* = 0.125 and *p* ≥ 0.07, respectively).

Finally, considering the total *Fusarium* fungal biomass (calculated as the sum of the fungal biomasses of each of the six *Fusarium* species analyzed) detected in durum wheat grains by qPCR, significant differences between cultivation areas were detected. In detail, the total *Fusarium* fungal biomass followed the significant gradient (*p* ≤ 0.04) Emilia Romagna >> Umbria > Sardinia.

### 2.4. Fungal Secondary Metabolites Accumulation

The fungal secondary metabolites (μg/kg) as quantified by LC/MS-MS in the durum wheat grains collected in the three Italian regions are summarized by category in Table 1 and detailed within each group in Table 2 (trichothecenes), Table 3 (zearalenone and fumonisins), Table 4 (depsipeptides), Table 5 (other *Fusarium* secondary metabolites), Table 6 (*Alternaria* secondary metabolites) and Table 7 (*Claviceps* secondary metabolites).

In general, samples collected in Emilia Romagna were particularly contaminated by *Fusarium* secondary metabolites (Table 1). Total trichothecenes followed the significant gradient (*p* ≤ 0.05) Emilia Romagna >> Umbria > Sardinia (Table 1). Total depsipetides followed the significant gradient (*p* ≤ 0.03) Emilia Romagna > Umbria = Sardinia (Table 1). The other *Fusarium* secondary metabolites followed the significant gradient (*p* ≤ 0.04): Emilia Romagna > Umbria > Sardinia (Table 1). The other two classes of compounds biosynthesized by *Fusarium*, zearalenone and fumonisins, were present in a low percentage of samples only in Emilia Romagna (Table 1 and Table 3). Conversely, *Alternaria* secondary metabolites were particularly present in durum wheat grains harvested in Umbria (Table 1). In detail, total *Alternaria* secondary metabolites accumulation followed the significant gradient (*p* ≤ 0.04): Umbria > Emilia Romagna > Sardinia. Finally, regarding *Claviceps* metabolites, even if they were mainly present in the Umbrian samples, no significant differences (*p* ≥ 0.26) between the three analyzed regions were detected, being only a few samples positive to these compounds (Table 1 and Table 7).

Focusing on trichothecenes (Table 2), LC-MS/MS analysis showed that DON, culmorin, 15-hydroxyculmorin, 15-hydroxyculmoron and 5-hydroxyculmorin were particularly present in durum wheat grains harvested in Emilia Romagna. In particular, DON followed the significant gradient (*p* ≤ 0.02) Emilia Romagna >> Umbria = Sardinia. A similar gradient (Emilia Romagna > Umbria = Sardinia) was also observed for culmorin and 15-hydroxyculmorin (*p* ≤ 0.04 and 0.03, respectively). The secondary metabolites 15-hydroxyculmoron and 5-hydroxyculmorin were present only in samples harvested in Emilia Romagna. Other trichothecenes, present in all surveyed regions were NIV, HT-2 toxin, HT-2 glucoside and T-2 toxin. These compounds were present at higher levels in Emilia Romagna samples, however, no significant differences were detected between Emilia Romagna and Umbria samples. In fact, they followed the significant gradient (*p* ≤ 0.05) Emilia Romagna = Umbria > Sardinia. Finally, samples positive to the presence of deacetylneosolaniol, diacetoxyscirpenol, T-2 tetraol and monoacetoxyscirpenol were found only in the Emilia Romagna and Umbria samples and, despite the higher levels of these compounds recovered in Emilia Romagna, no significant differences were detected between regions (*p* ≥ 0.18).

With regard to depsipeptides (Table 4), the EN analogues analyzed in this study (enniatin A, ENA; enniatin A1, ENA1; enniatin B, ENB; enniatin B1, ENB1; enniatin B2, ENB2; enniatin B3, ENB3) and BEA were all particularly present in Emilia Romagna samples followed by Umbria and Sardinia samples. In general, EN analogues amounts were higher than BEA in all the analyzed regions. The EN analogues accumulated in durum wheat grains harvested in Emilia Romagna and Umbria following the gradient ENB1 > ENB > ENA1 > ENB2 > ENA > ENB3. In durum wheat harvested in Sardinia, EN analogues followed the gradient ENB1 > ENB > ENB2 > ENB3. Significant differences between cultivation areas for ENB, ENB1 and ENB2 were recorded. All these three analogues were significantly higher in the Emilia Romagna samples (*p* ≤ 0.05), while, despite the higher levels detected in the Umbrian samples, no significant differences (*p* ≥ 0.12) were recorded between Umbrian and Sardinian ones. The analogues ENA, ENA1 and ENB3 were present only in Emilia Romagna and Umbria without (ENA and ENA1) or with (ENB3) significant differences between these two cultivation areas (*p* = 0.27, *p* = 0.20 and *p* = 0.02, respectively). With respect to BEA, the accumulation of this secondary metabolite followed a significant gradient (*p* ≤ 0.03) Emilia Romagna > Umbria > Sardinia.

Regarding other *Fusarium* secondary metabolites (Table 5), they were all present in the grains harvested in Emilia Romagna with the exception of epiequisetin that was present in the Umbrian grains. In detail, several compounds (antibiotic Y, chlamidospordiol, fusarin C, fusarinolic acid, sambucinol) were detected only in the Emilia Romagna grains. Some others (aminodimethyloctadecanol, aurofusarin, butenolide, chalmydosporol, epiequisetin and equisetin) were only present in the Emilia Romagna and Umbrian grains with significant differences recorded only for aurofusarin (*p* = 0.02). The remaining *Fusarium* metabolites (MON, apicidin and chrysogin) were detected in the grains from all surveyed regions, with significant differences found for MON (*p* ≤ 0.02, Emilia Romagna > Umbria = Sardinia) and chrysogin (*p* ≤ 0.003, Emilia Romagna > Umbria = Sardinia).

With respect to *Alternaria* secondary metabolites (Table 6), several compounds (tenuazonic acid, tentoxin, pyrenophorol, macrosporin) were notably higher in the Umbrian grains, while some others (altersetin, alternariol, infectopyrone and alternariol methyl ether) were notably higher in the Emilia Romagna samples. However, no significant differences were recorded between Umbria and Emilia Romagna for the content of these single compounds. Conversely, significant differences between these two regions and Sardinia were detected for tenuazonic acid, alternariol methyl ether and infectopyrone (Umbria = Emilia Romagna > Sardinia, *p* ≤ 0.04).

Finally, legislated mycotoxins did not exceed the maximum or recommended levels [12,13]. However, two samples harvested in Emilia Romagna showed DON levels (1740 and 1710 μg/kg) close to the maximum permitted for durum wheat grains (1750 μg/kg) and one sample collected in the same region showed T2+HT2 toxins level (97.4 μg/kg) close to the recommended concentration for durum wheat (100 μg/kg).

### 2.5. Correlation between Fungal Biomass and Fungal Secondary Metabolites in the Grains

With regard to *F. poae*, a typical BEA and NIV producer, its biomass was positively related to the amounts of BEA in the kernels, both for the Emilia Romagna and Umbrian samples, with correlation coefficients respectively of 0.72 (*p* = 0.018; *n* = 10) and 0.81 (*p* = 0.0048; *n* = 10). Likewise, *F. poae* biomass was positively correlated with the amounts of NIV in the kernels, only for the Umbrian samples (correlation coefficient = 0.79; *p* = 0.0071; *n* = 10)). Similarly, for *F. avenaceum*, one of the main ENs and MON producers, its biomass was positively related to the amounts of ENs (calculated by the sum of all six analyzed analogues) as well as with MON in the kernels of the Umbrian samples with correlation coefficients of 0.92 (*p* = 2 × 10^−4^; *n* = 10) and 0.93 (*p* = 1 × 10^−4^, *n* = 10), respectively. With respect to *F. graminearum*, a DON producer, its biomass was positively related to DON accumulation in the Emilia Romagna and Umbrian grains with correlation coefficients of 0.80 (*p* = 0.05, *n* = 10) and 0.95 (*p* = 1 × 10^−4^, *n* = 10), respectively. Finally, the sum of *F. sporotrichioides* and *F. langsethiae* biomasses, two of the main T-2 and HT-2 toxin producers, were positively correlated with the content of these two mycotoxins in the samples collected in Emilia Romagna and Umbria, with correlation coefficients of 0.95 (*p* = 1 × 10^−4^, *n* = 10) and 0.87 (*p* = 0.01, *n* = 10), respectively.

### 2.6. Weather Conditions in the Three Surveyed Regions

**Figure 4:**
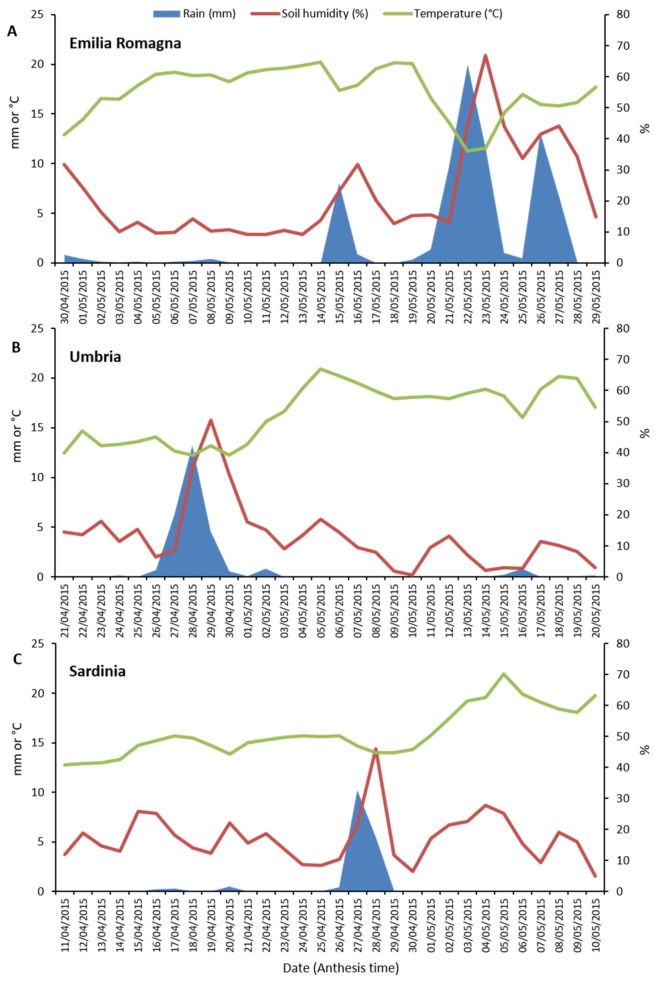
Weather data (rainfall, mm; soil humidity, %; average temperature, °C) recorded in the three surveyed Italian regions (Emilia Romagna, Umbria and Sardinia) during durum wheat anthesis phase. For each region, a period of thirty days during which anthesis usually occurs was considered. (**A**), Emilia Romagna. (**B**), Umbria. (**C**), Sardinia.

Weather data (rainfall, soil humidity and temperature) collected in the three different Italian regions (Emilia Romagna, Umbria and Sardinia) during the anthesis phase of durum wheat are shown in Figure 4. The highest rainfall levels (sum and daily average) during the considered 30-day period for each of the three regions, were recorded in Emilia Romagna (75.3 mm and 2.5 mm), followed by Umbria (27.7 mm and 0.9 mm) and then Sardinia (17.2 mm and 0.5 mm). In particular, in Emilia Romagna, heavy rainfalls occurred between 20 and 27 May. Differently, in the Umbria region, significant rain events were recorded between 26 and 30 April. Finally, in Sardinia, significant rain events occurred only on 27 and 28 April, indicating that in this region the anthesis phase was characterized by particularly dry conditions. Similarly, the highest soil humidity average level was detected in Emilia Romagna with a peak of 66.9% on the day after (23 May) the main rain event (22 May) which occurred in this region during crop anthesis.

## 3. Discussion

Climate is an important driver of different aspects of fungal biogeography, including global distribution of common fungi as well as composition and diversity of fungal communities [57]. Thus, this paper reports the results of a study about fungal communities and their secondary metabolites in durum wheat grains harvested in 2015 in three Italian regions (Emilia Romagna, Umbria and Sardinia) characterized by three different climatic conditions.

The mycobiota composition of durum wheat grains was investigated with two of the most common methods used for the isolation of seed-borne fungal pathogens worldwide (PDA and DFB) [58]. With regard to the global presence of *Fusarium* species, no differences between the two methods were detected, while, in these experimental conditions, the DFB method seemed to favor the development of microorganisms belonging to the genus *Alternaria*, probably due to the absence of grain external disinfection. Conversely, PDA appeared to favor the development of *Epicoccum*, *Aspergillus* and *Penicillium* species.

Species of the genus *Alternaria* were the main components of durum wheat mycobiota in all the investigated regions, showing their ubiquity across Italian cultivation areas. In Umbria, the prevalence of this genus in the durum wheat fungal community has been already highlighted [7]. Similarly, the high abundance of *Alternaria* species in wheat grains was previously detected also in other countries worldwide [9,55,59,60,61,62,63,64]. In fact, *Alternaria* species could be associated with wheat grains as saprophytes, causing black (sooty) head mold [65], or as pathogens, causing black point of kernels [66]. In addition, these species are potentially able to biosynthesize mycotoxins [6].

In this study, different incidence levels of this genus and of its related secondary metabolites in the three cultivation areas were detected, suggesting that environmental conditions may have influenced the presence of this fungal genus and of its mycotoxins. In particular, durum wheat samples harvested in Umbria (Central Italy) showed the highest levels of *Alternaria* species as well as, in general, of their secondary metabolites. Some *Alternaria* mycotoxins have been previously reported in durum wheat grains harvested in the Umbria region [40], however this study, expanding the number of compounds analyzed, revealed that tenuazonic acid, alternariol, alternariol methyl ether, altersetin, tentoxin, infectopyrone, macrosporin and pyrenophorol were widespread in this cultivation area. The presence of all or some of the analyzed *Alternaria* secondary metabolites in Emilia Romagna and Sardinia, even if with different levels, revealed the remarkable diffusion of these compounds in Italian durum wheat. Further studies will be necessary to identify the *Alternaria* species associated with durum wheat grains as well as to better understand the toxicity of *Alternaria* mycotoxins. In fact, despite their presence in cereals has been documented for years, no regulation has been applied so far worldwide, apart from the limits for tenuazonic acid recently established by the Bavarian Health and Food Safety Authority in infant food in Germany [6].

*Fusarium* species were the second most prevalent components of durum wheat mycobiota in all three cultivation environments. Previous studies have shown the presence of *Fusarium* spp. in the wheat grains fungal community globally [9,25,59,61,62,67,68,69,70] as well as in several Italian regions, including those investigated in this research [7,8,31,33,71] as well as in others [8,18,19,32]. In detail, Infantino et al. [32] and Shah et al. [18] showed that *Fusarium* incidence decreased from Northern to Southern Italian areas. Also in this study, differences among the three cultivation areas were observed. In general, *Fusarium* incidence was higher in Emilia Romagna followed by Umbria and then Sardinia, showing that durum wheat cultivated in Northern Italy might be exposed to a higher FHB infection risk, followed by Central Italy. This suggests that environmental conditions at the regional level (macroscale) play a remarkable effect on *Fusarium* incidence in durum wheat grains, also confirming that the gradual expansion of durum wheat from Southern to Northern areas of Italy might have increased the risks of FHB infections [4]. However, Scala et al. [19] suggested that environmental conditions at the local level (microscale) might drive FHB outbreaks even in areas suitable for growing durum wheat, such as Southern Italy. One of the reasons that may also have contributed to these regional differences (Emilia Romagna > Umbria > Sardinia) is represented by the rainfalls recorded during the anthesis phase of the crop (Figure 4). In fact, concerning the season investigated in this research, the rainfall levels occurred in the three surveyed regions followed the same gradient as of *Fusarium* infections. However, in association with climatic parameters, also other factors, such as crop rotation, fungicide application and cultivated varieties [49,51] may have played a role in *Fusarium* incidence in wheat grains.

In this study, the different *Fusarium* species were identified by partial *tef1α* region sequencing. With regard to the used isolation method (PDA or DFB), *F. proliferatum* growth was particularly promoted by DFB with respect to PDA. The lower *F. proliferatum* development on PDA might have been caused by the sterilization process applied before seed plating as well as by the quicker development of other fungal species. For this reason, the use of both isolation methods could be recommended to obtain a realistic indication of the *Fusarium* community associated with grains. In fact, the choice of a specific isolation method rather than another could lead to the underestimation or the overestimation of the incidence of one or more *Fusarium* species.

With respect to the detected *Fusarium* spp., each of the three examined regions was characterized by the presence of a specific complex of different species. In general, a predominance of *F. poae*, in particular in Emilia Romagna and Umbria, was recorded. Earlier surveys conducted in Italy showed the prevalence of this species in several growing seasons [8,18,32,33], particularly those characterized by unfavorable climatic conditions to the most damaging FHB agents, such as *F. graminearum*, at anthesis [31]. In fact, in Umbria, *F. poae* was the most frequently detected species in malting barley grains in 2014 [72] and, together with *F. avenaceum* [7,73], they seemed to temporarily replace *F. graminearum* as the main components of the FHB complex associated with wheat and barley. In addition, this study shows that, for the analyzed year and particularly based on PDA isolations, *F. poae* was the dominant species of the FHB complex in durum wheat grains harvested in the three different examined Italian regions. *F. avenaceum*, at least in Emilia Romagna and Umbria, never exceeded, in the examined year, *F. poae*. However, in Umbria, *F. avenaceum* was present at similar levels as of *F. graminearum*. This is a further confirmation of what previously observed in the Umbria region over the last few years, where *F. poae* and *F. avenaceum* seemed to interchange as the dominant members of the *Fusarium* community associated with durum wheat and malting barley, while, *F. graminearum* showed a constant incidence, never acting as the main species of the complex [7,72,73,74]. Interestingly, particularly based on DFB isolations, *F. proliferatum* showed a high incidence in the FHB complex of durum wheat grains harvested in all investigated regions. This species is typically associated with maize grains where it represents one of the ear rot causal agents, however, across the years, the occurrence of *F. proliferatum* in wheat has increased in many world cultivation areas [75] including Italy, and in particular in the Emilia Romagna region [76].

The regional differences observed for the incidence of the genus *Fusarium* was also recorded for the incidence of the single members within the complex. In particular, *F. poae*, *F. graminearum* and *F. proliferatum* were all extensively present in the Northern area followed by Central and finally Southern areas. Noteworthy is the fact that *F. graminearum* was totally absent in the samples from Sardinia. This highlights, as observed in other world cultivation areas [30,52,54,77], that also in Italy the environment plays an important role in the FHB complex composition both under quantitative (incidence of some species within the complex) and qualitative (presence/absence of certain species within the complex) points of view.

To further explore the FHB complex composition, the fungal biomass of six selected main *Fusarium* species was quantified directly in grains by qPCR. This method confirmed that the total *Fusarium* fungal biomass (sum of the fungal biomasses of each of the six species analyzed) as well as *F. poae* biomass were found to be higher in the Northern area, followed by Central and, finally, Southern areas. The absence of *F. graminearum* in the samples harvested in Sardinia was also confirmed by qPCR. Interestingly, *F. langsethiae* was detected by qPCR in all the investigated regions, while, it was isolated with both methods only in the grains harvested in Umbria and with a low incidence. This may have happened because *F. langsethiae*, a fungus characterized by a slow growth rate, might have been overgrown during the isolation process by other more rapidly growing species (such as *F. poae*) [38]. Therefore, to have a complete overview of the FHB complex composition of a certain cultivation area, the use of methods able to molecularly detect and quantify the different species directly in the grains, such as qPCR, may also be used. By contrast, the adoption of species-specific primers, such as those used in this study, may limit the detection to a few species only. As a consequence, the simultaneous adoption of qPCR assays for in grains fungal biomass quantification as well as of isolation methods coupled to *tef1α* region sequencing of the obtained *Fusarium* isolates, even if it is a time-consuming procedure, allows more realistic information to be obtained about the *Fusarium* community associated with durum wheat grains in a specific cultivation area.

Finally, as reported for other mycotoxigenic fungi [78] the observed regional differences in *Fusarium* incidence and FHB composition have important consequences for the quality of raw materials of derived products as well as for final consumers’ health.

In fact, the fungal secondary metabolites associated with durum wheat grains broadly reflected, with a few exceptions, the FHB complex composition obtained with both the use of the two isolation methods and qPCR assays. Besides, durum wheat grains harvested in the Northern area were more contaminated by *Fusarium* mycotoxins than those of Central and Southern areas. In particular, probably due to the higher presence of *F. graminearum*, trichothecenes, and DON among them, were particularly present in the grains harvested in Emilia Romagna. These samples also showed the highest BEA and ENs levels, possibly because of the highest *F. poae* and *F. avenaceum* presence, respectively. This highlights that, also in Italy, regional differences, as detected in other world areas [49,77], have a marked influence on the final *Fusarium* mycotoxin contamination. Surprisingly, despite the significant *F. proliferatum* presence, very low fumonisin levels (compared to those recovered in maize by Covarelli et al. [79]) were detected (a very low percentage of samples only in Emilia Romagna). Low levels of fumonisin contamination on wheat infected with *F. proliferatum* were also recorded in other wheat-cultivation areas [74,80].

## 4. Conclusions

In conclusion, characterizing the spatial dynamics of fungal communities, including the FHB complex, and of fungal secondary metabolites associated with durum wheat harvested in three different Italian regions, demonstrated that significant regional differences might occur. The Northern Italian cultivation area showed the highest levels of *Fusarium* incidence and in particular of *F. poae* and *F. graminearum* species. Therefore, the grains harvested in this area showed the highest *Fusarium* mycotoxins levels. The Central Italian cultivation area showed, in general, lower *Fusarium* incidence and *Fusarium* mycotoxins levels, but the highest level of *Alternaria* mycotoxins was detected in this area. The Southern Italian area, in the examined year, was shown to be the durum wheat cultivation environment where both *Fusarium* species and mycotoxins were present at the lowest levels, with positive repercussions on raw-material quality and on final consumers’ health.

## 5. Materials and Methods

### 5.1. Durum Wheat Sampling

**Figure 5:**
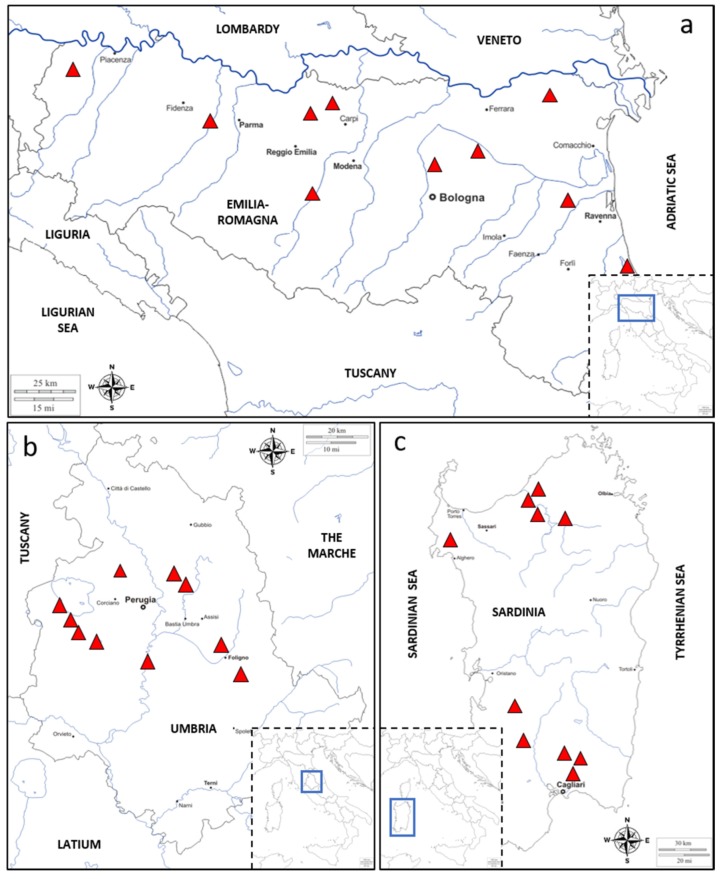
Maps of the three Italian regions representative of the three different durum wheat cultivation areas. In detail: map of the Emilia Romagna region (Northern Italy) (**a**), map of the Umbria region (Central Italy) (**b**), map of the Sardinia region (Southern Italy) (**c**) showing sampling locations (red triangles) and neighboring regions and/or seas.

This investigation was carried out on a total of 30 durum wheat samples collected from three Italian regions (10 samples per region) representative of three different Italian durum wheat cultivation areas (Figure 5a–c). The regions were Emilia Romagna (Northern Italy; Figure 5a), Umbria (Central Italy; Figure 5b) and Sardinia (Southern Italy; Figure 5c). All the samples were obtained from crops cultivated during the 2014–2015 season. The sampling strategy aimed at covering the most important areas of the three investigated regions (Figure 5a–c). Region of cultivation, cultivation site and variety for each durum wheat sample are indicated in Table S4. After harvest, samples (about 500 g each) were divided into three representative sub-samples of about 150 g each: one used for mycobiota determination, one for fungal biomass quantification directly in the grain by qPCR of six *Fusarium* species associated with FHB and one for fungal secondary metabolites analysis by LC-MS/MS.

### 5.2. Determination of Durum Wheat Mycobiota in the Grains

The mycobiota infecting durum wheat grains were detected with fungal isolations on PDA and with the DFB method.

#### 5.2.1. Isolation on Potato Dextrose Agar (PDA)

The isolation of the fungal community infecting durum wheat grains on PDA were performed as described by Beccari et al. [72]. In brief, about 30 g of the sub-sampled kernels were externally sterilized for 2 min using a water-ethanol (95%, Sigma Aldrich, Saint Louis, MO, USA)-sodium hypochlorite (7%, Carlo Erba Reagents, Milan, Italy) solution (82:10:8% vol.) and rinsed with sterile water for 1 min. After the sterilization process, 100 kernels were placed onto PDA (Biolife Italiana, Milan, Italy) supplemented with streptomycin sulphate (0.16 g/L, Sigma Aldrich) into 10 Petri dishes (90 mm diameter, Nuova Aptaca, Asti, Italy) containing 10 kernels each. The dishes were incubated at 22 °C in the dark and after 5 days of incubation a combination of visual and stereomicroscope (SZX9, Olympus, Tokyo, Japan) observations were carried out on each single kernel (10 kernels per replicate, 10 replicates per sample) to assess fungal development. The number of colonies (*n*) ascribable to the different fungal genera developed from the kernels collected in each of the three investigated regions was reported as the average (±standard error, SE) of the 10 samples.

#### 5.2.2. Isolation with the Deep-Freezing Blotter (DFB) Method

The isolation of the fungal community infecting durum wheat grains with the DFB method were realized as described by Limonard [81] with slight modifications. In brief, from about 30 g of sub-sampled kernels, one-hundred kernels were randomly collected and placed onto three sterilized layers of filter paper (90 mm diameter, grade 1) (Whatman, GE Healthcare, Amersham Place, UK) supplemented with 7 mL of sterile deionized water into 10 Petri dishes (100 mm diameter, Nuova Aptaca) containing 10 kernels each. After 24 h at 24 °C under near-ultraviolet (NUV) light, the dishes were placed at −20 °C for 24 h to devitalize germinating seeds. Finally, the dishes were incubated at 24 °C under NUV light and after 7 days of incubation a combination of visual and stereomicroscope (SZX9, Olympus, Tokyo, Japan) observations were carried out on each single kernel (10 kernels per replicate, 10 replicates per sample) to assess fungal development. The number of colonies (*n*) belonging to different fungal genera and developed from durum wheat kernels collected in each of the three investigated regions was reported as the average (±SE) of the 10 samples.

### 5.3. Molecular Identification of Fusarium spp. Isolated from Durum Wheat Grains

After visual observations, all the isolates potentially belonging to the genus *Fusarium* were transferred into new plates containing PDA and placed at 22 °C in the dark. After 10 days, *Fusarium* cultures developed from each single durum wheat sample were assigned to a particular morphotype according to colony color and shape on PDA by visual examination as well as to the morphology of reproductive structures by microscope analysis (Axiophot, Zeiss, Oberkochen, Germany). This selection allowed the obtainment of a subset of isolates composed of representative isolates of each morphotype for each durum wheat sample. These representative isolates, after obtaining monosporic cultures, were placed into new PDA plates at 22 °C in the dark for 2 weeks. Successively, their mycelium was scraped from PDA and placed into 2 mL sterile plastic tubes (Eppendorf, Hamburg, Germany) at −80 °C, freeze-dried with a lyophilizer (Heto Powder Dry LL3000; Thermo Fisher Scientific, Waltham, MA, USA) and the mycelium finely ground with a grinding machine (Retsch MM60, Dusseldorf, Germany) for 6 min with a frequency of 25 Hz.

DNA extraction was carried out using the method previously described by Covarelli et al. [31] with slight modifications described in Beccari et al. [72]. Extracted genomic DNA was visualized on a 1% agarose, trizma base-glacial acid acetic-ethylenediamine-tetraacetic acid disodium salt dehydrate (TAE; all from Sigma Aldrich) gel in TAE buffer (1×) containing 500 μL/L of RedSafe (iNtRON Biotechnology, Burlington, MA, USA). DNA fragments were separated in 10 cm-long agarose gels, with an electrophoresis apparatus (Eppendorf) applying a tension of 110 V for ~30 min. Electrophoretic runs were visualized using an ultraviolet transilluminator (Euroclone, Milan, Italy). DNA concentration was estimated by comparison with Gene Ruler 1 kb (Thermo Scientific Scientific) included in each gel as a control. DNA was diluted in DNase free sterile water for molecular biology use (5prime, Hilden, Germany) to obtain a concentration of ~30 ng/μL and stored at −20 °C until used.

The DNA extracted from *Fusarium* isolates was subject to *tef1α* gene amplification, purification and sequencing. A PCR protocol was adopted using a total reaction volume of 50 μL. Each reaction contained 29 μL of sterile water for molecular biology use, 5 μL of 10× Dream Taq Buffer + magnesium chloride (Thermo Fisher Scientific), 3.75 μL of cresol red (Sigma Aldrich), 5 μL of dNTP mix 10 mM (Microtech, Naples, Italy), 2.5 μL of 10 μM EF1 (ATGGGTAAGGA(A/G)GACAAGAC) and EF2 (GGA(G/A)GTACCAGT(G/C)ATCATGTT) primers [82,83], 0.25 μL of 5 U/μL Dream Taq Polymerase (Thermo Fisher Scientific) and 2 μL of template DNA. The PCR cycle consisted of an initial denaturation step (94 °C for 5 min), followed by 30 cycles of denaturation (94 °C for 1 min), annealing (53 °C for 1 min), extension (72 °C for 1 min), and of final extension (72 °C for 10 min). PCR fragments were purified and sequenced by an external sequencing service (Genewiz Genomics Europe, Takeley, United Kingdom). The sequence obtained were verified by Chromatogram Explorer Lite v4.0.0 (Heracle Biosoft srl 2011, Arges, Romania) and analyzed by the BLAST database [84].

PCR assays were performed on a T-100 thermal cycler (Bio Rad, Hercules, CA, USA). PCR fragments were visualized on TAE 1× agarose gel (2%) containing 500 μL/L of RedSafe. DNA fragments were separated by an electrophoresis apparatus applying a tension of 110 V for ~40 min. Electrophoretic runs were observed with an ultraviolet transilluminator. The size of the amplified fragments was obtained by comparison with HyperLadder 100–1000 bp (Bioline Meridian Bioscience, Cincinnati, OH, USA).

The presence of each *Fusarium* species involved in the FHB complex in each of the three investigated areas and for each isolation methods was calculated as the total number of isolates belonging to the morphotype from which the identified isolate was sub-sampled. The number of isolates (*n*) belonging to the different *Fusarium* species developed from durum wheat kernels collected in each of the three investigated regions was reported as the average (±SE) of the 10 samples for each isolation method.

### 5.4. Quantification of Fungal Biomass by Quantitative Real-Time Polymerase Chain Reaction (qPCR) in Durum Wheat Grains

To determine the standard curves for qPCR analysis, DNA was extracted from healthy durum wheat grains and from pure fungal cultures of *F. graminearum* (strain UK1, provided by John Innes Centre, Norwich, UK), *F. culmorum* (strain Fu42, provided by John Innes Centre, Norwich, UK), *F. avenaceum* (strain Fu71, provided by John Innes Centre, Norwich, UK), *F. langsethiae* (strain ER1400, provided by Council for Agricultural Research and Agricultural Economy Analysis, Rome, Italy), *F. poae* (strain Fu448, provided by University of Bologna, Bologna, Italy) and *F. sporotrichioides* (strain IT7637, provided by the Institute of Sciences of Food Production, National Research Council, Bari, Italy). All fungal strains were grown on PDA for one week prior to DNA extraction. Mycelium was scraped with a spatula, placed in a 2-mL sterile plastic tube (Eppendorf) at −80°C and freeze-dried by a lyophilizer (Heto Powder Dry LL3000, Thermo Fisher Scientific). Successively, the mycelium was finely ground with a grinding machine (Retsch MM60) for 6 min with a frequency of 25 Hz. DNA extraction was carried out using the method previously described by Covarelli et al. [31] with slight modifications described in Beccari et al. [72].

Durum wheat uncontaminated grains were finely ground in a blender (IMETEC, Azzano S. Paolo, Italy) and DNA extracted with the method described by Parry and Nicholson [85] with slight modifications described in Beccari et al. [86].

The concentration of extracted DNA was measured by a Qubit^®^ 2.0 fluorimeter (Thermo Fisher Scientific) using a Qubit^®^ dsDNA BR assay (molecular probes, Thermo Fisher Scientific) and following the manufacturer’s protocol. Dilution series from 0.5 pg to 5 ng of six fungal strains DNA, with a serial dilution factor of 10, were produced to set up standard curves that were processed in each qPCR assay. Two replicates of each standard were used in each assay. Standard curves were generated by plotting the logarithmic values of known DNA quantities versus the corresponding Ct values. For each standard curve, from the average Ct of each dilution, the line equation (*y = mx + q*) was calculated as well as the R^2^ value and the reaction efficiency (10^(−1/m)^). The limit of detection (LOD) of fungal biomass was 0.5 pg. Sub-sampled durum wheat kernels were finely ground with a blender and 2 g of ground grains were added into a 50 mL plastic tube (Thermo Fisher Scientific). Total DNA from the milled samples, including durum wheat DNA and potentially fungal DNA, was extracted using the method previously described by Parry and Nicholson [85] with slight modifications described in Beccari et al. [86]. Concentration of extracted DNA was estimated as previously described and the concentration of each DNA sample was adjusted to 20 ng/μL for qPCR analysis. Specific primers (Table S5) [87,88] were used for the quantification of six *Fusarium* species that might have potentially infected durum wheat kernels in the three investigated areas according to previous surveys conducted in the three regions on soft wheat, durum wheat and malting barley [7,31,33,70,71,73]. *Tef1α* primers were used for the quantification of durum wheat DNA (Table S5) [88].

To optimize qPCR analysis conditions, annealing temperatures (from 55 °C to 65 °C) were adjusted. qPCR assays were realized in a CFX96 Real-Time System (Bio-Rad). The qPCR reaction was composed of a total reaction volume of 12 μL, containing 2.5 μL of total DNA, 6 μL of 2× SYBR Select Master Mix for CFX (Applied Biosystem, Foster City, CA, USA), 1.5 μL of 2 μM of each primer and 0.5 μL of sterile DNase free water (Thermo Fisher Scientific). The cycle consisted of temperatures of 50 °C for 2 min, 95 °C for 10 min, 45 cycles at 95 °C for 15 sec and the specific annealing temperatures of each primer (Table S5 for 1 min, heating at 95 °C for 10 sec, cooling at 60 °C and finally an increase to 95 °C at 0.5 °C every 5 sec with the measurement of fluorescence. A dissociation curve was included at the end of the qPCR program to monitor the presence of primer-dimers and/or non-specific amplification products. Two replicates per each sample were used in each assay. The fungal biomass of each investigated *Fusarium* species, possibly present in the durum wheat grain was expressed as the ratio of the detected fungal DNA (pg) to the total durum wheat grain DNA (ng).

### 5.5. Detection and Quantification of Fungal Secondary Metabolites by Liquid Chromatography Tandem Mass Spectrometry (LC-MS/MS)

Sub-sampled durum wheat kernels were finely ground to <0.5 mm by a blender (IMETEC) and 5 g of each milled sample were extracted for 90 min on a rotary shaker (GFL3017, GFL, Burgwedel, Germany) using 20 mL extraction solvent (acetonitrile-water-acetic acid, 79:20:1, v/v/v). Raw extracts were diluted 1 + 1 using acetonitrile-water-acetic acid 20:79:1 (v/v/v) and 5 µl were subsequently injected. The instrumental method used in this study is an extension of the version described in detail by Malachova and co-authors [89]. Briefly, a QTrap 5500 MS/MS system (Sciex, Foster City, CA, USA) equipped with a TurboV electrospray ionization (ESI) source was coupled to a 1290 series ultra-high performance liquid chromatography (UHPLC) system (Agilent Technologies, Waldbronn, Germany). Chromatographic separation was performed at 25 °C on a Gemini C18-column, 150 × 4.6 mm i.d., 5 μm particle size, equipped with a C18 security guard cartridge, 4 × 3 mm i.d. (both Phenomenex, Torrance, CA, USA). Quantification was performed using external calibration based on serial dilution of a multi-analyte stock solution. Results were corrected using apparent recoveries that were determined for wheat by spiking experiments. The accuracy of the method is verified on a continuous basis by participation in a proficiency testing scheme organized by BIPEA (Gennevilliers, France) with a current success rate (i.e., a z-score between −2 and 2) of >94% of the >1100 results submitted and for 92 of the 95 results submitted for wheat, respectively.

### 5.6. Weather Data Collection

Weather data (rainfalls, soil humidity and temperatures) were obtained through the national network operated by the Italian Department of Civil Protection [90]. In detail, for each surveyed region (Emilia Romagna, Umbria and Sardinia) a period of thirty days during which anthesis usually occurs in the different durum wheat varieties was taken into account.

### 5.7. Statistical Analysis

Data about the mycobiota composition were analysed by using a generalised linear model (GLM) with binomial error and logit link. Isolation method and region were used as the factors. Data about the abundance of *Fusarium* species were analysed by a GLM, with Poisson error and log-link. A scale parameter was included to account for overdispersion, wherever necessary. Back-transformed counts with delta standard errors were derived and reported in figures and tables. Data about biomass and secondary metabolites (for each sample) were analysed by using a heteroscedastic linear model, with a different variance per region (generalised least square (GLS) fitting, [91]). The area was included as a factor. Both for GLM and GLS fits, pairwise comparisons were performed by using the general procedure outlined in Bretz et al. [92]. The correlations between fungal biomass and fungal secondary metabolites were studied, for each environment, by using the Pearson correlation coefficient. All analyses were performed by using the R statistical environment [93], together with the packages ‘nlme’ [91], ‘emmeans’ [94].

## Figures and Tables

**Table 1:** Categories of secondary metabolites as detected by liquid chromatography tandem mass spectrometry in durum wheat grains harvested in three different Italian regions (Emilia Romagna, Umbria, Sardinia).

Categories of Secondary Metabolites *	Positive Samples (*n* = 10)	Incidence Positive Samples (%)	Average Positive Samples (μg/kg)		Total Average (μg/kg)	
***Fusarium* secondary metabolites**						
**Trichothecenes**						
Emilia Romagna	10	100	1326 **	(±541)	1326 ***	(±541)
Umbria	10	100	104	(±28.1)	104	(±28.1)
Sardinia	6	60	65.1	(±20.1)	39.1	(±15.7)
**Zearalenone**						
Emilia Romagna	5	50	10.9	(±4.42)	5.46	(±2.76)
Umbria	nd ^§^	nd	nd	- ^†^	nd	-
Sardinia	nd	nd	nd	-	nd	-
**Fumonisins**						
Emilia Romagna	2	20	33.3	(±19.6)	6.65	(±5.31)
Umbria	nd	nd	nd	-	nd	-
Sardinia	nd	nd	nd	-	nd	-
**Depsipetides**						
Emilia Romagna	10	100	323	(±115)	323	(±115)
Umbria	10	100	56.4	(±36.2)	56.4	(±36.2)
Sardinia	3	30	0.23	(±0.15)	0.06	(±0.05)
**Other *Fusarium* secondary metabolites**						
Emilia Romagna	10	100	730	(±217)	730	(±217)
Umbria	9	90	106	(±47.3)	95.1	(±43.6)
Sardinia	6	60	1.82	(±0.57)	1.10	(±0.44)
***Alternaria* secondary metabolites**						
Emilia Romagna	10	100	366	(±35.8)	366	(±35.8)
Umbria	10	100	701	(±155)	701	(±155)
Sardinia	10	100	205	(±19.4)	205	(±19.4)
***Claviceps* secondary metabolites**						
Emilia Romagna	2	20	56.7	(±53.2)	11.3	(±10.9)
Umbria	3	30	359	(±298)	107	(±94.4)
Sardinia	3	30	1.40	(±1.35)	0.42	(±0.40)

* Sum of all secondary metabolites belonging to different categories that among all those analyzed showed their presence in at least one durum wheat sample are shown; ** the value represents the average (±standard error) of positive samples of each region; *** the value represents the average (±standard error) of 10 replicates for each region; ^§^ nd: not detected; ^†^ standard error not calculated.

**Table 2:** *Fusarium* trichothecene secondary metabolites as detected by liquid chromatography tandem mass spectrometry in durum wheat grains harvested in three different Italian regions (Emilia Romagna, Umbria, Sardinia).

Secondary Metabolites	Positive Samples (*n* = 10)	Incidence Positive Samples (%)	Average Positive Samples (μg/kg)		Total Average (μg/kg)		Max Value (μg/kg)
***Fusarium* secondary metabolites**							
Trichothecenes *							
**Deoxynivalenol**							
Emilia Romagna	10	100	499 **	(±210)	499 ***	(±210)	1740
Umbria	3	30	51.8	(±25.7)	15.5	(±10.3)	103
Sardinia	3	30	26.2	(±5.30)	7.85	(±4.22)	34.6
**Nivalenol**							
Emilia Romagna	10	100	63.6	(±14.8)	63.6	(±14.8)	158
Umbria	10	100	35.1	(±15.0)	35.1	(±15.0)	161
Sardinia	2	20	17.3	(±3.85)	3.47	(±2.38)	21.2
**Culmorin**							
Emilia Romagna	10	100	314	(±142)	314	(±142)	1222
Umbria	7	70	23.8	(±8.89)	16.7	(±7.08)	74.8
Sardinia	4	40	30.5	(±5.87)	12.2	(±5.42)	44.6
**15-Hydroxyculmorin**							
Emilia Romagna	10	100	203	(±83.8)	203	(±83.8)	698
Umbria	4	40	23.7	(±12.3)	9.49	(±5.94)	59.7
Sardinia	5	50	28.5	(±7.77)	14.2	(±5.99)	50.7
**15-Hydroxyculmoron**							
Emilia Romagna	6	60	86.9	(±33.2)	52.2	(±75.4)	203
Umbria	nd ^§^	nd	nd	- ^†^	nd	-	nd
Sardinia	nd	nd	nd	-	nd	-	nd
**5-Hydroxyculmorin**							
Emilia Romagna	5	50	266	(±100)	133	(±64.0)	564
Umbria	nd	nd	nd	-	nd	-	nd
Sardinia	nd	nd	nd	-	nd	-	nd
**Deacetylneosolaniol**							
Emilia Romagna	3	30	41.4	(±7.99)	12.4	(±6.65)	51.2
Umbria	3	30	17.3	(±1.76)	5.18	(±2.67)	20.4
Sardinia	nd	nd	nd	-	nd	-	nd
**Diacetoxyscirpenol**							
Emilia Romagna	3	30	3.53	(±3.13)	1.06	(±0.97)	9.80
Umbria	1	10	0.70	-	0.07	(±0.07)	0.70
Sardinia	nd	nd	nd	-	nd	-	nd
**HT-2 Glucoside**							
Emilia Romagna	5	50	29.5	(±11.8)	14.7	(±7.43)	69.3
Umbria	8	80	6.30	(±2.21)	10.7	(±3.62)	32.5
Sardinia	1	10	6.60	-	0.66	(±0.66)	6.60
**HT-2 toxin**							
Emilia Romagna	6	60	30.0	(±12.1)	17.9	(±8.52)	78.8
Umbria	8	80	13.5	(±3.99)	10.7	(±3.62)	32.5
Sardinia	2	20	2.10	(±1.48)	0.42	(±0.28)	2.10
**T-2 toxin**							
Emilia Romagna	4	40	9.35	(±3.47)	3.74	(±1.98)	18.6
Umbria	6	60	5.23	(±1.92)	3.14	(±1.39)	12.1
Sardinia	1	10	2.00	-	0.20	(±0.20)	2.00
**T2-Tetraol**							
Emilia Romagna	3	30	13.1	(±5.62)	3.94	(±2.47)	23.5
Umbria	3	30	4.20	(±0.60)	1.26	(±0.66)	5.40
Sardinia	nd	nd	nd	-	nd	-	nd
**Monoacetoxyscirpenol**							
Emilia Romagna	8	80	9.57	(±4.31)	7.66	(±3.63)	39.5
Umbria	4	40	5.00	(±2.05)	2.00	(±1.10)	11.1
Sardinia	nd	nd	nd	-	nd	-	nd

* Trichothecenes that among all those analyzed were present in at least one durum wheat sample; ** the value represents the average (±standard error) of positive samples for each region; *** the value represents the average (±standard error) of 10 replicates for each region; ^§^ nd: not detected; ^†^ standard error not calculated.

**Table 3:** *Fusarium* zearalenone and fumonisin secondary metabolites as detected by liquid chromatography tandem mass spectrometry in durum wheat grains harvested in three different Italian regions (Emilia Romagna, Umbria, Sardinia).

Secondary Metabolites	Positive Samples (*n* = 10)	Incidence Positive Samples (%)	Average Positive Samples (μg/kg)		Total Average (μg/kg)		Max Value (μg/kg)
***Fusarium* secondary metabolites**							
Zearalenone *							
**Zearalenone-sulfate**							
Emilia Romagna	4	40	15.5 **	(±3.91)	5.07 ***	(±2.59)	23.9
Umbria	nd ^§^	nd	nd	- ^†^	nd	-	nd
Sardinia	nd	nd	nd	-	nd	-	nd
**Zearalenone**							
Emilia Romagna	4	40	0.98	(±0.31)	0.39	(±0.20)	1.8
Umbria	nd	nd	nd	-	nd	-	nd
Sardinia	nd	nd	nd	-	nd	-	nd
Fumonisins *							
**Fumonisin B1**							
Emilia Romagna	2	20	30.3	(±16.5)	6.05	(±4.73)	46.8
Umbria	nd	nd	nd	-	nd	-	nd
Sardinia	nd	nd	nd	-	nd	-	nd
**Fumonisin B2**							
Emilia Romagna	1	10	6.04	-	0.60	(±0.60)	6.04
Umbria	nd	nd	nd	-	nd	-	nd
Sardinia	nd	nd	nd	-	nd	-	nd

* Zearalenone and fumonisin compounds that among all those analyzed were present in at least one durum wheat sample; ** the value represents the average (±standard error) of positive samples for each region; *** the value represents the average (±standard error) of 10 replicates for each region; ^§^ nd: not detected; ^†^ standard error not calculated.

**Table 4:** *Fusarium* depsipeptide secondary metabolites as detected by liquid chromatography tandem mass spectrometry in durum wheat grains harvested in three different Italian regions (Emilia Romagna, Umbria, Sardinia).

Secondary Metabolites	Positive Samples (*n* = 10)	Incidence Positive Samples (%)	Average Positive Samples (μg/kg)		Total Average (μg/kg)		Max Value (μg/kg)
***Fusarium* secondary metabolites**							
Depsipeptides *							
**Enniatin A**							
Emilia Romagna	8	80	5.30 **	(±3.71)	4.24 ***	(±3.00)	30.8
Umbria	6	60	1.16	(±0.32)	0.70	(±0.26)	2.20
Sardinia	nd ^§^	nd	nd	- ^†^	nd	-	nd
**Enniatin A1**							
Emilia Romagna	7	70	66.0	(±39.2)	46.2	(±28.6)	296
Umbria	5	50	12.3	(±3.45)	6.16	(±2.61)	23.9
Sardinia	nd	nd	nd	-	nd	-	nd
**Enniatin B**							
Emilia Romagna	9	90	137	(±32.9)	123	(±32.5)	319
Umbria	6	60	40.4	(±30.5)	24.2	(±18.8)	192
Sardinia	1	10	0.20	-	0.02	(±0.02)	0.20
**Enniatin B1**							
Emilia Romagna	9	90	148	(±56.5)	133	(±52.6)	564
Umbria	5	50	43.8	(25.3)	21.9	(±13.9)	144
Sardinia	1	10	0.30	-	0.03	(±0.03)	0.30
**Enniatin B2**							
Emilia Romagna	10	100	7.44	(±1.99)	7.44	(±1.99)	17.8
Umbria	5	50	2.80	(±2.13)	1.40	(±1.10)	11.3
Sardinia	1	10	0.10	-	0.01	(±0.01)	0.10
**Enniatin B3**							
Emilia Romagna	6	60	0.11	(±0.02)	0.07	(±0.02)	0.20
Umbria	1	10	0.10	-	0.01	(±0.01)	0.10
Sardinia	nd	nd	nd	-	nd	-	nd
**Beauvericin**							
Emilia Romagna	10	100	7.15	(±0.90)	7.15	(±0.90)	13.0
Umbria	10	100	1.95	(±0.88)	1.95	(±0.88)	9.60
Sardinia	1	10	0.10	-	0.01	(±0.01)	0.10

* Depsipeptides that among all those analyzed were present in at least one durum wheat sample; ** the value represents the average (±standard error) of positive samples for each region; *** the value represents the average (±standard error) of 10 replicates for each region; ^§^ nd: not detected; ^†^ standard error not calculated.

**Table 5:** Other *Fusarium* secondary metabolites as detected by liquid chromatography tandem mass spectrometry in durum wheat grains harvested in three different Italian regions (Emilia Romagna, Umbria, Sardinia).

Secondary Metabolites	Positive Samples (*n* = 10)	Incidence Positive Samples (%)	Average Positive Samples (μg/kg)		Total Average (μg/kg)		Max Value (μg/kg)
***Fusarium* secondary metabolites**							
Others *							
**Aminodimethyloctadecanol**							
Emilia Romagna	6	60	34.6 **	(±8.92)	20.7 ***	(±7.65)	67.0
Umbria	2	20	60.4	(±40.3)	12.1	(±10.1)	101
Sardinia	nd ^§^	nd	nd	- ^†^	nd	-	nd
**Antibiotic Y**							
Emilia Romagna	4	40	66.8	(±26.9)	26.7	(±14.6)	143
Umbria	nd	nd	nd	-	nd	-	nd
Sardinia	nd	nd	nd	-	nd	-	nd
**Moniliformin**							
Emilia Romagna	10	100	171	(±58.2)	171	(±58.2)	610
Umbria	5	50	48.4	(±32.8)	24.2	(±17.4)	177
Sardinia	2	20	0.80	(±0.70)	0.16	(±0.14)	1.50
**Apicidin**							
Emilia Romagna	8	80	47.9	(±31.6)	38.3	(±25.7)	249
Umbria	6	60	24.2	(±11.5)	14.5	(±7.72)	75.8
Sardinia	3	30	0.93	(±0.39)	0.28	(±0.17)	1.70
**Aurofusarin**							
Emilia Romagna	10	100	398	(±139)	398	(±139)	1400
Umbria	5	50	62.5	(±30.2)	31.2	(±17.6)	144
Sardinia	nd	nd	nd	-	nd	-	nd
**Butenolide**							
Emilia Romagna	2	20	46.6	(±28.2)	9.32	(±7.50)	74.8
Umbria	3	30	11.9	(±3.85)	3.57	(±2.07)	19.6
Sardinia	nd	nd	nd	-	nd	-	nd
**Chlamydospordiol**							
Emilia Romagna	2	20	6.85	(±0.15)	1.37	(±0.91)	7.00
Umbria	nd	nd	nd	-	nd	-	nd
Sardinia	nd	nd	nd	-	nd	-	nd
**Chlamydosporol**							
Emilia Romagna	4	40	16.8	(±5.29)	6.72	(±3.35)	30.9
Umbria	1	10	3.90	-	0.39	(±0.39)	3.90
Sardinia	nd	nd	nd	-	nd	-	nd
**Chrysogin**							
Emilia Romagna	10	100	17.4	(±4.72)	17.4	(±4.72)	41.6
Umbria	6	60	3.35	(±0.83)	2.01	(±0.72)	7.20
Sardinia	3	30	2.26	(±0.15)	0.68	(±0.34)	2.50
**Epiequisetin**							
Emilia Romagna	2	20	0.75	(±0.65)	0.15	(±0.13)	1.40
Umbria	4	40	0.90	(±0.42)	0.36	(±0.21)	2.10
Sardinia	nd	nd	nd	-	nd	-	nd
**Equisetin**							
Emilia Romagna	4	40	29.5	(±18.7)	11.8	(±8.35)	85.0
Umbria	9	90	7.46	(±2.62)	6.72	(±2.46)	26.8
Sardinia	nd	nd	nd	-	nd	-	nd
**Fusarin C**							
Emilia Romagna	3	30	11.6	(±2.66)	3.48	(±1.90)	16.6
Umbria	nd	nd	nd	-	nd	-	nd
Sardinia	nd	nd	nd	-	nd	-	nd
**Fusarinolic acid**							
Emilia Romagna	4	40	53.5	(±25.4)	21.4	(±12.7)	128
Umbria	nd	nd	nd	-	nd	-	nd
Sardinia	nd	nd	nd	-	nd	-	nd
**Sambucinol**							
Emilia Romagna	1	10	29.3	-	2.93	(±2.93)	29.3
Umbria	nd	nd	nd	-	nd	-	nd
Sardinia	nd	nd	nd	-	nd	-	nd

* Other *Fusarium* secondary metabolites that among all those analyzed were present in at least one durum wheat sample; ** the value represents the average (±standard error) of positive samples for each region; *** the value represents the average (±standard error) of 10 replicates for each region; ^§^ nd: not detected; ^†^ standard error not calculated.

**Table 6:** *Alternaria* secondary metabolites as detected by liquid chromatography tandem mass spectrometry in durum wheat grain samples harvested in three different Italian regions (Emilia Romagna, Umbria, Sardinia).

Secondary Metabolites	Positive Samples (*n* = 10)	Incidence Positive Samples (%)	Average Positive Samples (μg/kg)		Total Average (μg/kg)		Max Value (μg/kg)
*Alternaria* secondary metabolites *							
**Tenuazonic acid**							
Emilia Romagna	10	100	112 **	(±28.4)	112 ***	(±28.4)	321
Umbria	10	100	460	(±142)	460	(±142)	1450
Sardinia	10	100	44.3	(±12.9)	44.3	(±12.9)	155
**Alternariol**							
Emilia Romagna	5	50	1.50	(±0.78)	0.75	(±0.44)	4.50
Umbria	5	50	1.12	(±0.38)	0.56	(±0.26)	2.40
Sardinia	nd ^§^	nd	nd	- ^†^	nd	-	nd
**Alternariol methyl ether**							
Emilia Romagna	9	90	0.61	(±0.28)	0.55	(±0.25)	2.80
Umbria	10	100	0.74	(±0.43)	0.74	(±0.43)	4.50
Sardinia	1	10	0.20	^†^-	0.02	(±0.02)	0.20
**Altersetin**							
Emilia Romagna	10	100	36.6	(±11.9)	36.6	(±11.9)	113
Umbria	10	100	12.4	(±5.66)	12.4	(±5.66)	46.7
Sardinia	nd	nd	nd	-	nd	-	nd
**Tentoxin**							
Emilia Romagna	10	100	0.33	(±0.04)	0.33	(±0.04)	0.60
Umbria	10	100	0.83	(±0.35)	0.83	(±0.35)	3.90
Sardinia	6	60	0.47	(±0.20)	0.28	(±0.13)	1.40
**Infectopyrone**							
Emilia Romagna	10	100	214	(±15.2)	214	(±15.2)	296
Umbria	10	100	217	(±25.9)	217	(±25.9)	329
Sardinia	10	100	159	(±9.38)	159	(±9.38)	220
**Macrosporin**							
Emilia Romagna	10	100	1.00	(±0.37)	1.00	(±0.37)	3.90
Umbria	10	100	2.94	(±1.06)	2.94	(±1.06)	10.0
Sardinia	10	100	1.48	(±0.45)	1.48	(±0.45)	3.90
**Pyrenophorol**							
Emilia Romagna	1	10	8.00	-	0.80	(±0.80)	8.00
Umbria	5	50	11.8	(±5.29)	5.89	(±3.17)	32.3
Sardinia	nd	nd	nd	-	nd	-	nd

* *Alternaria* secondary metabolites that among all those analyzed were present in at least one durum wheat sample; ** the value represents the average (±standard error) of positive samples for each region; *** the value represents the average (±standard error) of 10 replicates for each region; ^§^ nd: not detected; ^†^ standard error not calculated.

**Table 7:** *Claviceps* secondary metabolites as detected by liquid chromatography tandem mass spectrometry in durum wheat grain samples harvested in three different Italian regions (Emilia Romagna, Umbria, Sardinia).

Secondary Metabolites	Positive Samples (*n* = 10)	Incidence Positive Samples (%)	Average Positive Samples (μg/kg)		Total Average (μg/kg)		Max Value (μg/kg)
*Claviceps* secondary metabolites *							
**Ergocornine**							
Emilia Romagna	nd ^§^	nd	nd	- ^†^	nd	-	nd
Umbria	2	20	13.3 **	(±9.43)	2.67 ***	(±1.98)	19.2
Sardinia	nd	nd	nd	-	nd	-	nd
**Ergocornin**							
Emilia Romagna	nd	nd	nd	-	nd	-	nd
Umbria	2	20	9.00	(±6.36)	1.80	(±1.31)	12.6
Sardinia	nd	nd	nd	-	nd	-	nd
**Ergocristine**							
Emilia Romagna	2	20	15.2	(±10.7)	3.04	(±2.95)	29.6
Umbria	nd	nd	nd	-	nd	-	nd
Sardinia	nd	nd	nd	-	nd	-	nd
**Ergocristinine**							
Emilia Romagna	2	20	9.25	(±6.54)	1.85	(±1.80)	18.1
Umbria	nd	nd	nd	-	nd	-	nd
Sardinia	nd	nd	nd	-	nd	-	nd
**Ergocryptine**							
Emilia Romagna	nd	nd	nd	-	nd	-	nd
Umbria	2	20	60.7	(±42.9)	12.1	(±10.9)	110
Sardinia	nd	nd	nd	-	nd	-	nd
**Ergocryptinine**							
Emilia Romagna	nd	nd	nd	-	nd	-	nd
Umbria	2	20	34.9	(±24.7)	6.99	(±6.27)	63.1
Sardinia	nd	nd	nd	-	nd	-	nd
**Ergometrine**							
Emilia Romagna	2	20	13.5	(±9.58)	2.71	(±2.49)	25.1
Umbria	2	20	145	(±102)	29.1	(±24.1)	29.1
Sardinia	1	10	4.10	-	0.41	(±0.41)	0.41
**Ergometrinine**							
Emilia Romagna	2	20	2.20	(±1.55)	0.44	(±0.40)	4.10
Umbria	2	20	24.5	(±17.3)	4.90	(±4.12)	41.4
Sardinia	1	10	0.10	-	0.01	(±0.01)	0.10
**Ergosin**							
Emilia Romagna	1	10	16.2	16.2	1.62	(±1.62)	16.2
Umbria	2	20	123	(±87.2)	24.7	(±21.6)	217
Sardinia	nd	nd	nd	-	nd	-	nd
**Ergosinin**							
Emilia Romagna	1	10	5.20	-	0.52	(±0.52)	5.20
Umbria	2	20	42.3	(±29.8)	8.45	(±7.34)	73.9
Sardinia	nd	nd	nd	-	nd	-	nd
**Ergotamine**							
Emilia Romagna	1	10	7.50	-	0.75	(±0.75)	7.50
Umbria	1	10	0.20	-	0.02	(±0.02)	0.20
Sardinia	nd	nd	nd	-	nd	-	nd
**Ergotaminine**							
Emilia Romagna	1	10	3.70	-	0.37	(±0.37)	3.70
Umbria	1	10	0.10	-	0.01	(±0.01)	0.10
Sardinia	nd	nd	nd	-	nd	-	nd
**Secalonic acid D**							
Emilia Romagna	nd	nd	nd	-	nd	-	nd
Umbria	1	10	165	-	16.5	(±16.5)	165
Sardinia	nd	nd	nd	-	nd	-	nd
**Agroclavine**							
Emilia Romagna	nd	nd	nd	-	nd	-	nd
Umbria	1	10	1.50	-	0.15	(±0.15)	1.50
Sardinia	nd	nd	nd	-	nd	-	nd
**Chanoclavin**							
Emilia Romagna	1	10	0.30	-	0.03	(±0.03)	0.30
Umbria	2	20	2.00	(±1.41)	0.40	(±0.36)	3.70
Sardinia	nd	nd	nd	-	nd	-	nd

* *Claviceps* secondary metabolites that among all those analyzed were present in at least one durum wheat sample; ** the value represents the average (±standard error) of positive samples for each region; *** the value represents the average (±standard error) of 10 replicates for each region; ^§^ nd: not detected; ^†^ standard error not calculated.

## Supplementary Materials

The following are available online at https://www.mdpi.com/2072-6651/12/2/97/s1: Table S1: Colonies belonging to the different fungal genera as visually and microscopically assessed after their development from durum wheat kernels collected in three different Italian regions (Emilia Romagna, Umbria, Sardinia) with two different isolation methods, Table S2: *Fusarium* species as identified by partial *translation elongation factor 1α* sequencing after their isolation with two different methods from durum wheat kernels collected in three different Italian regions (Emilia Romagna, Umbria, Sardinia), Table S3: Fungal biomass of six *Fusarium* species as quantified by quantitative real time polymerase chain reaction (qPCR) in durum wheat kernels collected in three different Italian regions (Emilia Romagna, Umbria, Sardinia), Table S4: Durum wheat samples analyzed in this study showing sample ID, growing region, sampling location and variety, Table S5: Primer sequences and characteristics used in real time quantitative polymerase chain reaction (qPCR) assays.
