# Supplementary material for: Cultivation Area Affects the Presence of Fungal Communities and Secondary Metabolites in Italian Durum Wheat Grains
Source: Toxins (Basel). 2020 Feb 3;12(2):97. doi: 10.3390/toxins12020097 (PMC7076967; doi:10.3390/toxins12020097)
Supplement: Supplementary file 1 [file toxins-12-00097-s001.zip › toxins-672154 supplementary 2/supplementary_files/toxins-672154-supplementary.docx]

Supplementary Materials: Cultivation Area Affects the Presence of Fungal Communities and Secondary Metabolites in Italian Durum Wheat Grains

Giovanni Beccari, Antonio Prodi *, Maria Teresa Senatore, Virgilio Balmas, Francesco Tini, Andrea Onofri, Luca Pedini, Michael Sulyok, Luca Brocca and Lorenzo Covarelli

**Table S1.** Colonies belonging to the different fungal genera as visually and microscopically assessed after their development from durum wheat kernels collected in three different Italian regions (Emilia Romagna, Umbria, Sardinia) with two different isolation methods.

| **Region** | **Method** | **Fungal Genera** | | | | | |
| --- | --- | --- | --- | --- | --- | --- | --- |
|  |  | ***Alternaria*** | ***Fusarium*** | ***Epicoccum*** | ***Aspergillus*** | ***Penicillium*** | **Other** |
| Emilia Romagna | PDA^†^ | 5.66 (±0.15)* | 2.67 (±0.10) | 0.88 (±0.08) | 0.11 (±0.03) | 0.11 (±0.03) | 0.81 (±0.08) |
|  | DFB^‡^ | 5.84 (±0.15) | 3.04 (±0.10) | nd^§^ | 0.42 (±0.06) | 0.20 (±0.04) | 0.68 (±0.07) |
| Umbria | PDA | 6.51 (±0.15) | 1.82 (±0.10) | 0.66 (±0.26) | 0.33 (±0.05) | 0.21 (±0.04) | 0.85 (±0.08) |
|  | DFB | 9.68 (±0.05) | 1.32 (±0.10) | nd | nd | nd | 0.66 (± 0.07) |
| Sardinia | PDA | 8.11 (±0.12) | 0.35 (±0.10) | 0.03 (±0.01) | 0.16 (±0.04) | 0.20 (±0.04) | 0.96 (±0.09) |
|  | DFB | 9.75 (±0.04) | 1.15 (±0.05) | nd | nd | nd | 0.13 (±0.03) |

^†^Potato dextrose agar; ^‡^Deep freezing blotter; *the value represents the average (± standard error) of 10 replicates; ^§^nd: not detected.

**Table S2.** *Fusarium* species as identified by partial *translation elongation factor 1α* sequencing after their isolation with two different methods from durum wheat kernels collected in three different Italian regions (Emilia Romagna, Umbria, Sardinia).

| **Region** | **Method** | ***Fusarium* Species*** | | | | | | | | | | | | | |
| --- | --- | --- | --- | --- | --- | --- | --- | --- | --- | --- | --- | --- | --- | --- | --- |
|  |  | **FP** | **FA** | **FG** | **FC** | **FS** | **FL** | **FT** | **FE** | **FAc** | **FPr** | **FV** | **FSa** | **FCr** |  |
| Emilia Romagna | PDA^†^ | 6.11 (±0.80)** | 0.51 (±0.23) | 3.01 (±0.56) | 0.56 (±0.24) | nd | nd | 0.18 (±0.13) | 0.20 (±0.13) | 0.10 (±0.07) | 0.84 (±0.29) | 0.15 (±0.12) | 0.20 (±0.14) | nd |  |
|  | DFB^‡^ | 4.33 (±0.65) | 0.67 (±0.25) | 2.82 (±0.52) | nd | nd | nd | 0.28 (±0.16) | 0.48 (±0.21) | 0.10 (±0.07) | 5.98 (±0.76) | 0.42 (±0.20) | nd | 0.10 (±0.07) |  |
| Umbria | PDA | 2.53 (±0.52) | 0.23 (±0.15) | 0.66 (±0.26) | 0.13 (±0.11) | 0.10 (±0.07) | 0.10 (±0.07) | 0.10 (±0.08) | nd | 0.10 (±0.07) | 0.67 (±0.26) | 0.10 (±0.08) | nd | nd |  |
|  | DFB | 1.55 (±0.40) | 0.53 (±0.23) | 0.87 (±0.30) | nd | nd | nd | 0.10 (±0.09) | 0.10 (±0.07) | 0.10 (±0.07) | 2.82 (±0.55) | nd | nd | nd |  |
| Sardinia | PDA | 0.26 (±0.16) | 0.10 (±0.07) | nd^§^ | 0.18 (±0.13) | nd | nd | nd | 0.10 (±0.08) | nd | nd | 0.10 (±0.07) | nd | nd |  |
|  | DFB | 0.13 (±0.12) | 0.10 (±0.09) | nd | 0.14 (±0.13) | 0.47 (±0.23) | nd | nd | 0.10 (±0.09) | nd | 0.92 (±0.33) | 0.10 (±0.09) | nd | nd |  |

*FP: *Fusarium poae*; FA: *Fusarium avenaceum*; FG: *Fusarium graminearum*; FC: *Fusarium culmorum*; FS: *Fusarium sporotrichioides*; FL: *Fusarium langsethiae*; FT: *Fusarium tricinctum*; FE: *Fusarium equiseti*; FAc: *Fusarium acuminatum*; FPr: *Fusarium proliferatum*; FV: *Fusarium verticillioides*; FSa: *Fusarium sambucinum*; FCr: *Fusarium crockwellense*; ^†^potato dextrose agar; ^‡^deep freezing blotter; **the value represents the average (± standard error) of 10 replicates; ^§^nd: not detected.

**Table S3.** Fungal biomass of six *Fusarium* species as quantified by quantitative real time polymerase chain reaction (qPCR) in durum wheat kernels collected in three different Italian regions (Emilia Romagna, Umbria, Sardinia).

| **Region** | ***Fusarium* Species* (pg of Fungal DNA/ng Durum Wheat Grains DNA)** | | | | | |
| --- | --- | --- | --- | --- | --- | --- |
|  | **FP** | **FA** | **FG** | **FL** | **FS** | **FC** |
| Emilia Romagna | 1.12 (±0.22)** | 0.76 (±0.16) | 1.20 (±0.71) | 0.16 (±0.10) | 0.010 (±0.01) | 0.03 (±0.01) |
| Umbria | 0.53  (±0.14) | 0.11 (±0.07) | 0.03 (±0.03) | 0.08 (±0.03) | nd | 0.01 (±0.01) |
| Sardinia | 0.17  (±0.09) | 0.01 (±0.01) | nd^§^ | 0.01 (±0.01) | nd | 0.21 (±0.09) |

*FP: *Fusarium poae*; FA: *Fusarium avenaceum*; FG: *Fusarium graminearum*; FL: *Fusarium langsethiae*; FS: *Fusarium sporotrichioides*; FC: *Fusarium culmorum*; **the value represents the average (± standard error) of 10 replicates; ^§^nd: not detected.

**Table S4.** Durum wheat samples analyzed in this study showing sample ID, growing region, sampling location and variety.

| **Sample ID** | **Region** | **Sampling Location** | **Variety** |
| --- | --- | --- | --- |
| 1 | Emilia Romagna | Noceto | PR22D78 |
| 2 | Emilia Romagna | Molinella | Achille |
| 3 | Emilia Romagna | Cervia | Tyrex |
| 4 | Emilia Romagna | Jolanda di Savoia | Obelix |
| 5 | Emilia Romagna | Argelato | Monastir |
| 6 | Emilia Romagna | Mezzano | Marakas |
| 7 | Emilia Romagna | Sassuolo | Odisseo |
| 8 | Emilia Romagna | Novellara | Orobel |
| 9 | Emilia Romagna | Reggiolo | Odisseo |
| 10 | Emilia Romagna | Sarmato | Orobel |
| 1 | Umbria | Casalina | Odisseo |
| 2 | Umbria | Ramazzano | Iride |
| 3 | Umbria | Ramazzano | Prospero |
| 4 | Umbria | Castiglione del Lago | Colorado |
| 5 | Umbria | Foligno | Avispa |
| 6 | Umbria | Spello | PR22D40 |
| 7 | Umbria | Colle Umberto | Dylan |
| 8 | Umbria | Bagnaia | Dylan |
| 9 | Umbria | Panicale | Claudio |
| 10 | Umbria | Panicarola | Dylan |
| 1 | Sardinia | Valledoria | Karalis |
| 2 | Sardinia | Valledoria | Iride |
| 3 | Sardinia | Sestu | Karalis |
| 4 | Sardinia | San Gavino | Karalis |
| 5 | Sardinia | Santa Maria Coghinas | Karalis |
| 6 | Sardinia | Ussana | Saragolla |
| 7 | Sardinia | Bachileddu | Saragolla |
| 8 | Sardinia | Samassi | Rusticano |
| 9 | Sardinia | Laerru | Karalis |
| 10 | Sardinia | Ussana | Karalis |

**Table S5.** Primer sequences and characteristics used in real time quantitative polymerase chain reaction (qPCR) assays.

| **Target** | **Primer Name** | **Sequences (5’–3’)** | **Annealing Temp. (°C)** | **References** |
| --- | --- | --- | --- | --- |
| *F. graminearum* | Fg16N F | ACAGATGACAAGATTCAGGCACA | 61 | [1] |
|  | Fg16N R | TTCTTTGACATCTGTTCAACCCA |  |  |
| *F. poae* | FpoaeA51 | ACCGAATCTCAACTCCGCTTT | 61 | [2] |
|  | FpoaeA98 | GTCTGTCAAGCATGTTAGCACAAGT |  |  |
| *F. avenaceum* | Fave574 | TATGTTGTCACTGTCTCACACCACC | 61 | [2] |
|  | Fave627 | AGAGGGATGTTAGCATGATGAAG |  |  |
| *F. culmorum* | FculC561 | CACCGTCATTGGTATGTTGTGACTGG | 61 | [2] |
|  | FculC614 | CGGGAGCGTCTGATAGTCG |  |  |
| *F. sporotrichioides* | FspoS18 | GCAAGTCGACCACTGTGAGTACA | 61 | [2] |
|  | FspoA85 | CTGTCAAAGCATGTCAGTAAAAATGAT |  |  |
| *F. langsethiae* | FlangA29 | CAAGTCGACCACTGTGAGTACCTCT | 61 | [2] |
|  | FlangA95 | TGTCAAAGCATGTCAGTAAAGATGAC |  |  |
| *Translation elongation factor 1α* | Hor1 F | TCTCTGGGTTTGAGGGTGAC | 60 | [2] |
|  | Hor2 R | GGCCCTTGTACCAGTCAAGGT |  |  |

References

1. Brandfass, C.; Karlovsky, P. Upscaled CTAB-Based DNA extraction and Real-Time PCR assays for *Fusarium culmorum* and *F. graminearum* DNA in plant material with reduced sampling error. *Int. J. Mol. Sci.* **2008**, *9*, 2306–2321.
2. Nicolaisen, M.; Supronien, S.; Nielsen, L.K.; Lazzaro, I.; Spliid, N.H.; Justesen, A.F. Real-time PCR for quantification of eleven individual *Fusarium* species in cereals*. J. Microbiol. Methods* **2009**, *76*, 234–240.
